# Breakfast Frequency and Smoking Initiation in University Students: A Retrospective Cohort Study

**DOI:** 10.3390/nu16142361

**Published:** 2024-07-21

**Authors:** Rika Mori, Ryohei Yamamoto, Maki Shinzawa, Naoko Otsuki, Yuichiro Matsumura, Yuko Nakamura, Qinyan Li, Yusuke Sakaguchi, Isao Matsui, Masayuki Mizui, Haruki Shinomiya, Chisaki Ishibashi, Kaori Nakanishi, Daisuke Kanayama, Izumi Nagatomo

**Affiliations:** 1 Laboratory of Behavioral Health Promotion, Department of Health Promotion, Graduate School of Medicine, Osaka University, Toyonaka 560-0043, Japan; mori-r@office.osaka-u.ac.jp (R.M.); matsumura@hacc.osaka-u.ac.jp (Y.M.); li_qinyan@hacc.osaka-u.ac.jp (Q.L.); 2 Division of Nursing, Osaka University Hospital, Suita 565-0871, Japan; 3 Health and Counseling Center, Osaka University, Toyonaka 560-0043, Japan; shinzawa@kid.med.osaka-u.ac.jp (M.S.); otsuki@hacc.osaka-u.ac.jp (N.O.); nakamura@hacc.osaka-u.ac.jp (Y.N.); shinomiya@cardiology.med.osaka-u.ac.jp (H.S.); k-nakanishi@hacc.osaka-u.ac.jp (K.N.); iznagatomo@hacc.osaka-u.ac.jp (I.N.); 4 Department of Nephrology, Graduate School of Medicine, Osaka University, Suita 565-0871, Japan; sakaguchi@kid.med.osaka-u.ac.jp (Y.S.); matsui@kid.med.osaka-u.ac.jp (I.M.); mmizui@kid.med.osaka-u.ac.jp (M.M.)

**Keywords:** breakfast frequency, smoking, university students, retrospective cohort study

## Abstract

Smoking causes various health problems. Limited studies have reported a clinical effect of skipping breakfast on smoking initiation among adolescents. This retrospective cohort study aimed to assess the dose-dependent association between skipping breakfast and smoking initiation in university students. This study included 17,493 male and 8880 female students aged 18−22 years at a national university in Japan. The association between breakfast frequency (eating every day and skipping occasionally, often, and usually) and smoking initiation was evaluated using Cox proportional hazards models adjusted for clinically relevant factors. Smoking initiation was observed in 2027 (11.6%) male and 197 (2.2%) female students over the median observational period of 3.0 and 3.1 years. Skipping breakfast was significantly associated with smoking initiation in a dose-dependent fashion in male students (the adjusted hazard ratios [95% confidence interval] of eating breakfast every day and skipping occasionally, often, and usually: 1.00 [reference], 1.30 [1.15, 1.46], 1.47 [1.21, 1.79], and 1.77 [1.40, 2.25], respectively). Female students skipping breakfast occasionally and often were more vulnerable to smoking initiation than those who ate breakfast every day (1.00 [reference], 1.86 [1.24, 2.78], 2.97 [1.66, 5.32], and 1.76 [0.55, 5.64], respectively). Breakfast frequency may be useful to identify university students at risk of smoking initiation who need improvement in their health literacy.

## 1. Introduction

Smoking tobacco causes various health problems, including stroke [1], heart failure [2], chronic kidney disease [3,4], cancers [5], chronic obstructive pulmonary disease [6], and early vision loss [7]. A recent increase in the prevalence of adolescent smokers has been regarded as an important global public health problem [8]. The Department of Health of the U.S. reported that nearly 80% of adult tobacco smokers started smoking before they turned 18 [9]. Early smoking initiation is regarded as a risk factor for a wide variety of health issues. A Cuban cohort study suggested that the childhood initiation of smoking nearly doubled the incidence of premature death in adulthood [10]. To develop an effective strategy to reduce new young smokers, it is essential to identify lifestyle risk factors for smoking initiation.

One potential lifestyle factor linked to smoking initiation could be breakfast frequency, besides short sleep duration [11] and overeating [12]. Multiple studies clarified that people who skipped breakfast were at a higher risk of obesity [13,14], hypertension [15,16], type 2 diabetes [17,18], chronic kidney disease [19,20], proteinuria [20], and cardiovascular disease [16] than those who did not. Few studies reported the clinical relevance of skipping breakfast to smoking among young adults. A Japanese retrospective cohort study reported that skipping breakfast was associated with smoking initiation in a dose-dependent fashion among 12,872 university students [21]. However, this interesting association was not verified separately among men and women in this study. An international survey, the Global Adult Tobacco Survey, reported that 48.6% and 11.3% of men and women were smokers [22]. Worldwide, the prevalence of smoking every day in 2015 was 25.0% and 5.4% among men and women, respectively [23]. Due to the great difference in the prevalence of smokers between men and women, the association between skipping breakfast and smoking initiation needs to be assessed in men and women separately. Additionally, sex-dependent confounding factors possibly affect the clinical relevance of skipping breakfast to smoking initiation. Excessive alcohol consumption is one of the major unhealthy lifestyles as well as skipping breakfast among university students. A retrospective cohort study of university students in Japan reported a sex-dependent association with skipping breakfast and frequent alcohol consumption [24]. The influence of skipping breakfast on frequent drinking was stronger among female students compared to male students who skipped breakfast [24]. These reports suggest that the clinical relevance of skipping breakfast to smoking initiation should be evaluated separately in men and women.

This retrospective cohort study, including 17,493 male and 8880 female university students at a national university in Japan, aimed to clarify a sex-specific dose-dependent association between skipping breakfast and smoking initiation over a 6-year observational period. The results of this study clarify the clinical relevance of skipping breakfast to smoking initiation in adolescents.

## 2. Materials and Methods

### 2.1. Participants

**Figure 1:**
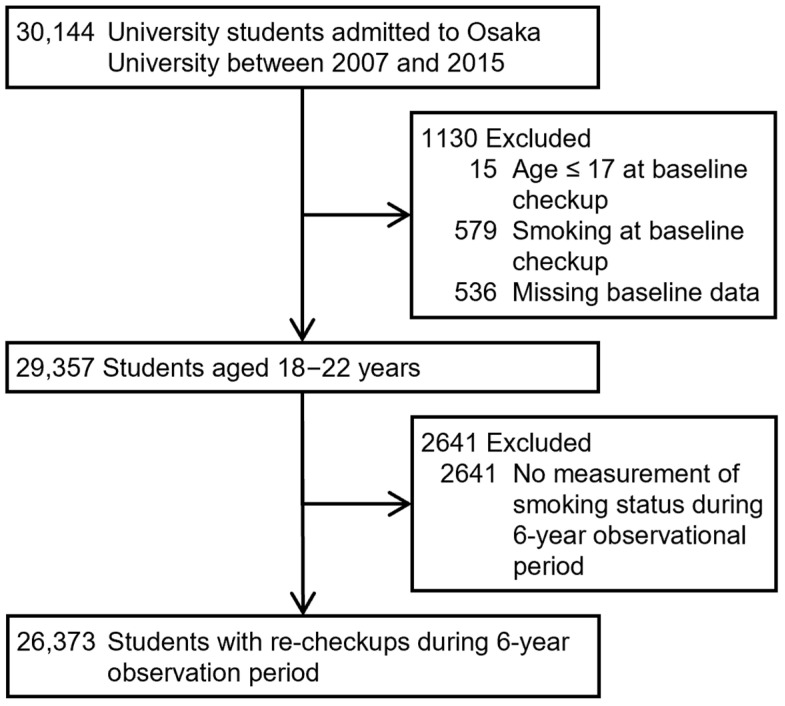
Flowchart depicting inclusion and exclusion of study participants.

A total of 30,144 university students who entered Osaka University, one of Japan’s largest national universities, between 2007 and 2015 were eligible for this study. These participants underwent baseline health checkups on admission in either April or October. We excluded 15 (0.1%) students aged ≤ 17 years on admission, 579 (1.9%) smokers at the baseline health checkups on admission, 536 (1.8%) students with missing baseline checkup data, and 2641 (8.8%) with no answer to the smoking question during the annual health checkups over the 6-year observation period after the baseline health checkups. Lastly, we included 26,373 (87.5%) students in the analysis to assess the association between skipping breakfast and smoking initiation (Figure 1).

The ethics committee of the Health and Counseling Center of Osaka University (No. 1 on 25 April 2024) and Osaka University Hospital (No. 18352-4 on 24 October 2022) approved the study protocol of this study. According to the Japanese Ethical Guidelines for Medical and Health Research Involving Human Subjects, an opt-out approach was employed to obtain informed consent in this study.

All the procedures involving human participants were conducted following the ethical guidelines set by both the institutional and national research committees, as well as the principles outlined in the 1964 Helsinki Declaration and its subsequent amendments or equivalent ethical guidelines.

### 2.2. Measurements

This study obtained all the data from the digital database maintained by the Health and Counseling Center at Osaka University. The baseline variables measured at the health checkups on admission included sex, age, body mass index (BMI = body weight [kg]/height^2^ [m^2^]), general questionnaires regarding breakfast frequency, smoking status, and potential confounders such as the frequency of alcohol consumption, sleep duration during weekdays, and living arrangements. The question “How often did you have breakfast during the past year?” measured breakfast frequency, for which the available answers were “eating almost every day”, “skipping occasionally”, “skipping often,” and “skipping usually”. Smoking status was evaluated using the question, “Do you smoke?” for which the available answers were “I do not smoke,” “I quit smoking”, “I would like to quit smoking”, and “I smoke”. Because the majority of students responded “I do not smoke” (*n* = 15,466 [88.4%] in men and 8683 [97.8%] in women), smoking status was categorized into two groups: non-smokers answering “I do not smoke.”, and smokers based on other responses. Alcohol consumption frequency was determined using the question “How often did you drink during the past year?” with four possible answers: “I did not drink”, “I drank occasionally”, “I drank a day/week”, and “I drank ≥ 2 days/week”. Sleep duration during weekdays was ascertained using the question “How long do you sleep on weekdays?” with five answers of <5, 5−6, 6−7, 7−8, and ≥ 8 h. Living arrangements were assessed with the question “What are your living arrangements?” offering choices such as living with family, living alone, living in a dormitory, and other living arrangements.

The outcome was the incidence of smoking initiation based on the answer to the smoking question at the annual health checkups after the baseline checkup during the 6-year (2190 days) observational period. The observational period was defined as the duration from the baseline checkup on admission until either the occurrence of the outcome or the most recent response to the smoking question at the annual health checkups within the 6-year observational period, whichever came first.

### 2.3. Statistical Analysis

To compare the baseline characteristics among the four categories of breakfast frequency, the analysis of variance (ANOVA) or χ^2^ test were used, as appropriate. The cumulative probability of smoking initiation was calculated using the Kaplan–Meier method. These probabilities were compared among the four groups of breakfast frequency using the log-rank test. To evaluate the association between breakfast frequency and smoking initiation, unadjusted and multivariable-adjusted Cox proportional hazard (CPH) models were used to calculate unadjusted and adjusted hazard ratios (HRs) with a 95% confidence interval (CI). The multivariable-adjusted models included age (18, 19, 20, 21, and 22 years), BMI (kg/m^2^), the frequency of alcohol consumption (never drinkers, occasional drinkers, current drinkers one day/week, and current drinkers ≥ 2 days/week), living arrangements (living with family, living alone, living in dormitories, and other living arrangements), and sleep duration during weekdays (<5, 5−6, 6−7, 7−8, and > 8 h) as covariates.

Continuous variables were expressed as either mean ± standard deviation or median (25th−75th percentile), as applicable. Categorical variables were reported as numbers with proportions. Statistical significance was defined at *p* ˂ 0.05. All the statistical analyses were conducted by Stata (version 17.0; Stata Corp., College Station, TX, USA).

## 3. Results

### 3.1. Baseline Characteristics

Table 1 shows the baseline characteristics of 17,493 male students. Most students (*n* = 14,214 [81.3%]) ate breakfast every day. Compared with men with higher breakfast frequency, men with lower breakfast frequency were more likely to be older, drink alcohol more frequently, and have a higher prevalence of both shorter and longer sleep duration. Among 8880 female students, eating breakfast every day was more prevalent (*n* = 7820 [88.1%]) than among men (Table 2). Women with lower breakfast frequency were more likely to be older and had a higher prevalence of both shorter and longer sleep duration compared to those with higher breakfast frequency (Table 2).

### 3.2. Skipping Breakfast and Smoking Initiation

**Figure 2:**
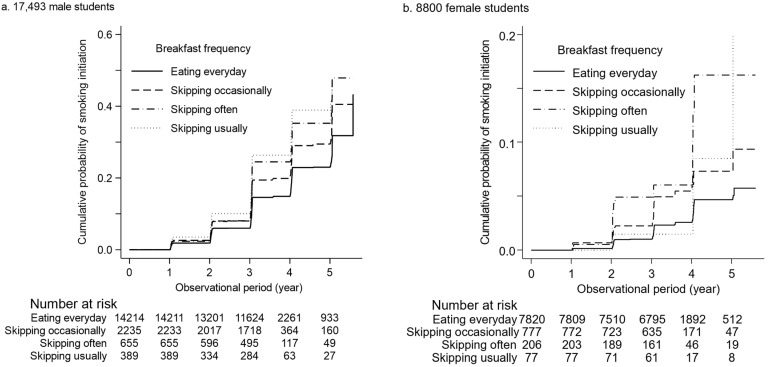
Baseline breakfast frequency and smoking initiation in 17,493 male (**a**) and 8880 female (**b**) students.

During the median observational period of 3.0 years (interquartile range 2.1−3.9), smoking initiation was observed in 1517 (10.7%), 325 (14.5%), 111 (16.9%), and 74 (19.0%) men who ate breakfast every day, skipped it occasionally, skipped it often, and skipped it usually, respectively (Table 3). Compared to men who ate breakfast every day, those who skipped breakfast occasionally, often, and usually had a significantly higher cumulative probability of smoking initiation (Figure 2). In an unadjusted model, skipping breakfast was associated with smoking initiation (unadjusted HR [95% CI] for eating every day, skipping occasionally, often, and usually: 1.00 [reference], 1.39 [1.23, 1.57], 1.57 [1.30, 1.90], and 1.89 [1.49, 2.38], respectively) (Table 3). The multivariable-adjusted models verified that men who skipped breakfast were at higher risk of smoking initiation than those who ate breakfast every day in a dose-dependent manner (adjusted HR [95% CI: 1.00 [reference], 1.30 [1.15, 1.46], 1.47 [1.21, 1.79], and 1.77 [1.40, 2.25], respectively).

Among female students, smoking initiation occurred in 151 (1.9%), 29 (3.0%), 14 (6.8%), and 3 (3.9%) students who ate breakfast every day and skipped it occasionally, often, and usually, respectively, during the median observational period of 3.0 years (interquartile range 2.1−3.9) (Table 3). The cumulative probability of smoking initiation was comparable among the four groups of breakfast frequency. An unadjusted model showed that skipping breakfast occasionally and often were significantly associated with the incidence of smoking initiation (unadjusted HR [95% CI]: 1.00 [reference], 2.05 [1.38−3.06], 3.57 [2.06−6.18], and 2.06 [0.66−6.45], respectively) (Table 3). The multivariable-adjusted models confirmed that those skipping breakfast sometimes and usually were at a significantly high risk of smoking initiation (adjusted HR [95% CI]: 1.00 [reference], 1.86 [1.24−2.78], 2.97 [1.66−5.32], and 1.76 [0.55−5.64], respectively) (Table 3).

To compare the risk for smoking initiation between male and female students, we calculated the unadjusted and adjusted HRs of the eight categories of sex × breakfast frequency among 17,493 male and 8880 female students after setting female students who ate breakfast every day as a reference (Table 3). The unadjusted and adjusted models showed that male students were significantly at higher risk for smoking initiation than female students.

## 4. Discussion

This retrospective cohort study showed that university students who skipped breakfast were at a higher risk for smoking initiation than those who ate breakfast every day. The main strengths of the present study include its large sample size of 26,373 university students (17,493 men and 8880 women) and its cohort study design with a 6-year observational period. These aspects facilitated a statistically robust evaluation of the impact of skipping breakfast on smoking initiation among both male and female students.

The findings of the present study were consistent with previous studies, including cross-sectional studies or retrospective cohort studies with smaller numbers of participants than that of the present study. A cross-sectional study of 19,542 school-aged adolescents in China reported that male and female students who skipped breakfast were more likely to smoke than those who had breakfast every day [25]. A retrospective cohort study, involving Japanese 12,872 university students, confirmed that students who skipped breakfast ≥ two times per week were more likely to start smoking compared to those who skipped breakfast ≤ once per week [21]. However, few cohort studies have evaluated the sex-dependent effect of skipping breakfast on smoking initiation. This retrospective cohort study, including 17,493 male and 8880 female students, showed that female students who skipped breakfast were more susceptible to smoking initiation (adjusted HR [95% CI]: 1.00 [reference], 1.86 [1.24, 2.78], 2.97 [1.66, 5.32], and 1.76 [0.55, 5.64], respectively) than male students (1.00 [reference], 1.30 [1.15, 1.46], 1.47 [1.21, 1.79], and 1.77 [1.40, 2.25], respectively) (Table 3).

Depression might be one plausible mechanism linking skipping breakfast to smoking initiation. Previous studies have suggested that skipping breakfast is associated with depression [25,26,27]. A Korean cross-sectional study of 153,992 adolescents aged 12–18 years reported that adolescents who skipped breakfast had a higher prevalence of depression compared to those who ate breakfast daily [28]. Adolescents with depression are more vulnerable to smoking. A systematic review identified depression as a predictor of some types of smoking behavior [29]. A cross-sectional study of 963 college students in Chile reported that smoking was associated with common mental disorders [30]. A Polish cross-sectional study of 316 junior high school students showed that adolescents with depressive symptoms based on the Beck Depression Inventory and Krakow Depression Inventory smoked more often than those without depressive symptoms, especially among girls [31]. The findings of these studies suggest that university students who skipped breakfast might have been more susceptible to depression and were more likely to start smoking. Additional research is crucial to investigate the underlying mechanisms that connect skipping breakfast to smoking initiation.

The present study has several limitations. First, this cohort study included university students only from a single university in Japan. The results of this study need to be verified at universities in different countries. Second, this study measured smoking status using self-reported questionnaires, which are susceptible to misreporting, rather than biological markers such as salivary cotinine. However, a previous study showed that self-reported questionnaires are comparable to salivary cotinine levels [32], suggesting the validity of this study. Third, the association between skipping breakfast and smoking initiation may have been influenced by unmeasured confounding factors, including exercise habits. A cross-sectional study in the United States showed that regular exercise was an important protective factor against smoking [33]. A Japanese study that included 1083 elementary school students reported that children eating breakfast every day were more prone to participate in regular sports activities than those who did not [34]. These results suggest that university students who skip breakfast and have low exercise activity might be susceptible to smoking initiation. Additional research is needed to identify the association between exercise activity and smoking initiation among university students. The main strengths of our study include its large sample size of over 26,000 participants and the robust assessment of the sex-specific association between skipping breakfast and smoking initiation, which could enhance the reliability of our findings.

## 5. Conclusions

This retrospective cohort study of 17,493 male and 8880 female students from a single national university in Japan found that skipping breakfast was a significant predictor of smoking initiation. These findings suggest that breakfast frequency may be useful to identify university students at risk of smoking initiation. University students who skip breakfast may be potential candidates who need improvement in health literacy, leading to the prevention of smoking initiation. A well-planned study is crucial to confirm the clinical effect of skipping breakfast on smoking initiation among university students.

## Figures and Tables

**Table 1:** Baseline characteristics of 17,493 male university students.

	Baseline Breakfast Frequency			
	Eating Every Day	Skipping Occasionally	Skipping Often	Skipping Usually
Number	14,214	2235	655	389
Age, 18 years, *n* (%)	9450 (66.5)	1315 (58.8)	351 (53.6)	223 (57.3)
19	4407 (31.0)	804 (35.9)	252 (38.5)	135 (34.7)
20	253 (1.8)	77 (3.5)	29 (4.4)	9 (2.31)
21	104 (0.7)	39 (1.7)	23 (3.5)	22 (5.7)
Body mass index, kg/m^2^	21.6 ± 2.9	21.5 ± 2.9	21.2 ± 2.9	21.3 ± 3.0
Never drinkers, *n* (%)	13,099 (92.2)	1943 (86.9)	543 (82.9)	331 (85.1)
Occasional drinkers	954 (6.7)	234 (10.5)	92 (14.1)	46 (11.8)
Current drinkers, 1 day/week	80 (0.6)	26 (1.2)	8 (1.2)	5 (1.3)
≥2 days/week	81 (0.6)	32 (1.4)	12 (1.8)	7(1.8)
Sleep duration, ≤5 h, *n* (%)	420 (3.0)	70 (3.1)	29 (4.4)	21 (5.4)
5–6	4546 (32.0)	698 (31.2)	193 (29.5)	131 (33.7)
6–7	6957 (48.9)	1059 (47.4)	287 (43.8)	155 (39.9)
7–8	2014 (14.2)	320 (14.3)	114 (17.4)	59 (15.2)
≥8	277 (1.9)	88 (3.9)	32 (4.9)	23 (5.9)
Living with family, *n* (%)	6990 (49.2)	936 (41.9)	282 (43.1)	191 (49.1)
Living alone	6177 (43.5)	1107 (49.5)	315 (48.1)	172 (44.2)
Living in dormitory	861 (6.1)	157 (7.0)	53 (8.1)	19 (4.9)
Other living arrangements	186 (1.3)	35 (1.6)	5 (0.8)	7 (1.8)

Mean ± standard deviation; percentages are shown in parentheses (%).

**Table 2:** Baseline characteristics of 8880 female university students.

	Baseline Breakfast Frequency			
	Eating Every Day	Skipping Occasionally	Skipping Often	Skipping Usually
Number	7820	777	206	77
Age, 18 years, *n* (%)	5852 (74.8)	539 (69.4)	119 (57.8)	49 (63.6)
19	1794 (22.9)	191 (24.6)	55 (26.7)	20 (26.0)
20	111 (1.4)	23 (3.0)	17 (8.3)	4 (5.2)
21	63 (0.8)	24 (3.1)	15 (7.3)	4 (5.2)
Body mass index, kg/m^2^	20.5 ± 2.4	20.3 ± 2.5	20.6 ± 2.4	19.9 ± 2.3
Never drinkers, *n* (%)	7513 (96.1)	716 (92.2)	172 (83.5)	70 (90.9)
Occasional drinkers	284 (3.6)	54 (6.9)	26 (12.6)	6 (7.8)
Current drinkers, 1 day/week	11 (0.1)	3 (0.4)	6 (2.9)	0 (0.0)
≥2 days/week	12 (0.2)	4 (0.5)	2 (1.0)	1 (1.3)
Sleep duration, ≤5 h, *n* (%)	214 (2.7)	35 (4.5)	13 (6.3)	5 (6.5)
5–6	2795 (35.7)	276 (35.5)	66 (32.0)	26 (33.8)
6–7	3767 (48.2)	339 (43.6)	82 (39.8)	32 (41.6)
7–8	955 (12.2)	111 (14.3)	36 (17.5)	9 (11.7)
≥8	89 (1.1)	16 (2.1)	9 (4.4)	5 (6.5)
Living with family, *n* (%)	4132 (52.8)	354 (45.6)	92 (44.7)	42 (54.6)
Living alone	2970 (37.9)	339 (43.6)	98 (47.6)	28 (36.4)
Living in dormitory	538 (6.9)	67 (8.6)	11 (5.3)	6 (7.8)
Other living arrangements	180 (2.3)	17 (2.2)	5 (2.4)	1 (1.3)

Mean ± standard deviation; percentages are shown in parentheses (%)

**Table 3:** Baseline breakfast frequency and smoking initiation in male and female students.

	Eating Every Day	Skipping Occasionally	Skipping Often	Skipping Usually
17,493 male students				
Smoking initiation, n (%)	1517 (10.7)	325 (14.5)	111 (16.9)	74 (19.0)
Observational period, year	3.0 ± 0.9	2.9 ± 0.9	3.0 ± 1.0	2.9 ± 1.0
IR per 1000 PY (95% CI)	35.4 (33.7, 37.3)	49.4 (44.3, 55.0)	57.3 (47.5, 68.6)	66.6 (53.0, 83.6)
Unadjusted HR (95% CI)	1.00 (reference)	1.39 (1.23, 1.57)	1.57 (1.30, 1.90)	1.89 (1.49, 2.38)
Adjusted HR (95% CI)*	1.00 (reference)	1.30 (1.15, 1.46)	1.47 (1.21, 1.79)	1.77 (1.40, 2.25)
8880 female students				
Smoking initiation, n (%)	151 (1.9)	29 (3.7)	14 (6.8)	3 (3.9)
Observational period, year	3.1 ± 0.8	3.0 ± 0.9	3.0 ± 1.0	3.0 ± 1.0
IR per 1000 PY (95% CI)	6.07 (5.18, 7.12)	12.2 (8.4, 17.5)	22.2 (13.2, 37.4)	12.6 (4.1, 39.1)
Unadjusted HR (95% CI)	1.00 (reference)	2.05 (1.38, 3.06)	3.57 (2.06, 6.18)	2.06 (0.66, 6.45)
Adjusted HR (95% CI)*	1.00 (reference)	1.86 (1.24, 2.78)	2.97 (1.66, 5.32)	1.76 (0.55, 5.64)
17,493 male and 8880 female students				
Unadjusted HR (95% CI)				
Male students	6.23 (5.27, 7.36)	8.65 (7.1, 10.49)	9.78 (7.65, 12.5)	11.7 (8.89, 15.5)
Female students	1.00 (reference)	2.05 (1.38, 3.05)	3.57 (2.06, 6.17)	2.04 (0.65, 6.39)
Adjusted HR (95% CI)				
Male students	5.65 (4.73, 6.74)	7.30 (6.0, 8.94)	8.29 (6.43, 10.7)	9.95 (7.48, 13.2)
Female students	1.00 (reference)	1.92 (1.29, 2.86)	3.41 (1.97, 5.91)	2.06 (0.66, 6.48)

Mean ± standard deviation. CI, confidence interval; HR, hazard ratio; IR, incidence rate; PY, person-year; *Adjusted for age (18, 19, 21, and 22 years), body mass index (kg/m^2^), frequency of alcohol consumption (never drinkers, occasional drinkers, current drinkers 1 day/week, and current drinkers ≥ 2 days/week), living arrangements (living with family, living alone, living in dormitories, and other living arrangements), and sleep duration during weekdays (<5, 5–6, 6–7, 7–8, and ≥ 8 h).

## Data Availability

The data presented in this study are available upon request from the corresponding author. The data are not publicly available because they were not originally collected for research purposes.
